# The Thermography Analysis of the Fine-Grained Materials Agglomeration Process in Closed Dies

**DOI:** 10.3390/ma18163796

**Published:** 2025-08-13

**Authors:** Andrzej Uhryński, Dariusz Lepiarczyk, Filip Matachowski, Michał Bembenek

**Affiliations:** 1 Department of Machine Design and Maintenance, Faculty of Mechanical Engineering and Robotics, AGH University of Krakow, A. Mickiewicza 30, 30-059 Krakow, Poland; uhrynski@agh.edu.pl (A.U.); filip.matachowski@agh.edu.pl (F.M.); 2 Department of Manufacturing Systems, Faculty of Mechanical Engineering and Robotics, AGH University of Krakow, A. Mickiewicza 30, 30-059 Krakow, Poland

**Keywords:** briquette, thermography, die, fine-grained materials, densification process, agglomeration process

## Abstract

A new method of diagnostics of technological parameters of the material agglomeration process and a design of a new matrix for compacting fine-grained materials were presented. Two different dies were used in the agglomeration process to carry out the tests. One of them is a new prototype design of an opening matrix, which allows for its quick opening and closing and easier extraction of the briquette and measurement. Comparative tests were carried out for four materials with different chemical compositions. The experimental part showed changes in the temperature of samples subjected to agglomeration. Analysis of the results for the Electric Arc Furnace Dust (EAFD) mixture showed that the average temperature in the middle of the material was higher by 1.3 °C than the highest temperature on the external surface. Comparing the test results for the hydrated lime obtained in the opening matrix with the results obtained for the classic closed matrix, it can be seen that the results are very similar, which indicates the advantage of the prototype solution of the opening matrix. The prototype solution of the opening matrix significantly speeds up the agglomeration process. The obtained results confirm the possibility of using thermography diagnostics in the agglomeration processes of fine-grained materials and the usefulness of the newly designed die. Thermography examination can be practically used to control the agglomeration process, product quality, and, indirectly, roller wear.

## 1. Introduction

The agglomeration process of fine-grained materials in dies has been the subject of scientific research for many years, particularly in increasing energy efficiency, environmental protection, improving the mechanical properties of finished products, and optimizing manufacturing processes [1]. Agglomeration of fine-grained materials in a closed die involves transforming loose material into briquettes by applying pressure that causes particles to come closer together and tighten their arrangement. This process takes place in the closed space of the die, allowing for high density and the production of briquettes with the desired shape and strength [2,3].

The agglomeration process uses various fine-grained materials such as biomass, coal, metallurgical waste, and ceramic raw materials [4,5]. Biomass briquetting is gradually becoming a method for sustainable energy production [6,7]. Interest in briquetting stems from the continuous rise in energy costs and the need for efficient and low-cost alternatives [8]. Briquetting has evolved and now includes mixing biomass with animal and municipal waste such as manure, microalgae, plastics, sludge, and food waste [9]. The wide availability of biomass materials in many regions worldwide has made this process practical and inexpensive [10].

Briquetting technology has gained acceptance in the scientific community as a way to achieve a circular and eco-friendly economy. In recycling, briquetting enables the compression of waste into solid blocks, facilitating their transport, storage, and further processing. This process is used to recycle metals, plastics, and industrial waste, reducing their volume and improving recovery efficiency [11]. Briquetting is also used in blast furnace production processes [12].

New manufacturing technologies, methods, and structural solutions have recently been developed to increase agglomeration process efficiency. Hydraulic and mechanical presses with gravity or screw feeders are used in the agglomeration process [13,14]. Various die designs are used for agglomeration. Dies with a movable matrix are also used in hot briquetting technology for structurally heterogeneous metallic waste. This solution improves the quality and efficiency of the pressing process [15]. To increase the reliability of the briquetting process, the design of a screw feeder has been presented [16]. The screw feeder increases pressing pressure, allowing agglomeration of materials that are otherwise difficult to briquette. Technologies using ultrasound are also applied, which help reduce friction and increase the uniformity of agglomeration [17].

Computer simulations optimize the agglomeration process, allowing the prediction of briquettes’ mechanical and physical properties without costly experiments [18]. Using machine learning methods, compression force, moisture content, compressive strength, briquette density, water resistance, cracking index, and compressive stress can be simulated [19].

Various physical and chemical phenomena affect the compacted material during the agglomeration process of fine-grained materials. These include particle adhesion and cohesion, plastic and elastic deformation, capillary phenomena, and especially thermal phenomena. Agglomeration technology is also crucial as it affects the density and strength of the briquettes [20]. Various testing and imaging methods are used to assess the quality of the agglomeration process [21,22], among which thermography deserves special attention.

Thermographic diagnostics is a process of imaging in the mid-infrared band. It allows for registering thermal radiation emitted by any object with a temperature above absolute zero. Due to its remote and non-contact measurement advantages, thermography is used in many research areas [23,24]. Particularly noteworthy is the use of thermography in the diagnostics of mechanical properties, durability, strength, and the detection of defects in structural materials [25]. In medicine, thermography detects inflammation, tumors, retinal vessel segmentation, and other conditions [26,27].

Thermal diagnostics involves detecting and registering emitted infrared radiation and converting it into a visible image that reflects the temperature field associated with the examined surface. Infrared radiation is recorded using thermal imaging cameras, which allow for the visualization of the temperature distribution emitted by individual elements of the observed surface.

In the agglomeration process, thermography is applied by recording the intensity of thermal radiation emitted. The thermal image received from the thermal imaging camera represents the temperature field of the surface of the briquette or the die. The temperature increase is caused by internal friction and particle interaction during consolidation. Heat is generated between the particles of the consolidated material, changing the temperature within a specific volume of the briquette. The higher the pressure applied to the material during agglomeration, the greater the degree of agglomeration and the higher the consolidation temperature. Therefore, the temperature distribution on the surface of the briquettes may be associated with the local pressure applied.

Experiments described in work [28] showed that temperature measurement using thermography in the briquetting process may indirectly determine the degree of agglomeration of the briquette or the pressure generated in a roller press during consolidation. It may also be used to select the appropriate geometry for forming chambers and to evaluate their wear.

It was hypothesized that the agglomeration process of fine-grained materials can be effectively assessed using thermographic techniques both in a classic closed die and in a prototype opening die, and that the latter would prove more suitable for future research. Initial tests were planned for pressures of 20 MPa, 60 MPa, to 160 MPa. After analyzing the issue, reviewing preliminary test results, and considering data from previous studies [27,28], a decision was made to apply relatively high pressure and feed rate close to maximum values. The final values used were a pressure of 150 MPa and a 25 mm/min feed rate. The maximum pressures available on the strength testing machine were used because lower forces did not achieve sufficient agglomeration of the samples, which caused difficulties in removing them intact from the dies.

## 2. Experimental Section

### 2.1. Materials

Four mixtures were prepared for the experiment:

- Material M1—90.9% by mass of calcium hydroxide Ca(OH)_2_ manufactured by Lhoist according to EN 459-1 CL 90-S and 9.1% by mass of water. The mixture was thoroughly mixed for five minutes by hand
- Material M2—hydrated lime consisting of 100% calcium hydroxide Ca(OH manufactured by Lhoist according to EN 459-1 CL 90-S.
- Material M3—47.7% by weight of Electric Arc Furnace Dust (EAFD), 36.7% mill scale, 7.3% coke breeze, 2.8% hydrated lime (EN 459-1 CL 90-S), and 5.5% of a molasses-water mixture (57.9% molasses and 42.1% water by weight). The dry ingredients were mixed manually in one container and the molasses-water mixture in another. The solution was then gradually added to the dry mixture, stirring for five minutes.
- Material M4—96% charcoal and 4% starch.

From the prepared mixtures, briquette samples were formed weighing 5 g each.

### 2.2. Equipment

The agglomeration process was carried out using a universal testing machine. The press exerts a maximum compressive stress of 200 MPa with a die diameter of 20 mm, corresponding to the diameter of the formed briquette, and a maximum feed rate of 25 mm/min. Two dies were used for the agglomeration (pressure agglomeration) process. Both dies consisted of a punch and a casing.

**Figure 1:**
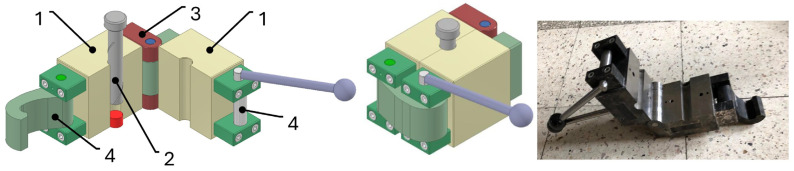
Closed opening die [29].

In the classic closed die, the material usually had to be extracted using the punch, which affected measurement accuracy due to prolonged extraction time and the resulting temperature change of the tested object. To solve this, a special opening die was constructed to eliminate this issue. An additional advantage of this solution is the shorter time required to extract the briquette, improving the accuracy of the temperature measurements. The construction of the new opening die is shown in Figure 1.

The die consists of two body parts (1 on Figure 1) that correctly align the opening for the punch (2 on Figure 1). The opening and closing of the die halves are achieved via hinges fastened to the rear part of the body (3 on Figure 1). A cam lock (4 on Figure 1) protects against unintentional openings during pressing. This lock consists of fastening elements attached with screws and a camshaft which, when rotated, locks the clamp placed on it. The clamp and pin form the components of the lock.

Opening and closing the die using the cam lock significantly shortens the briquette production process. An extra pin is also used to lift the working chamber relative to the base to position the punch opening. This opening die design significantly accelerates post-consolidation measurements, minimizing possible measurement errors.

### 2.3. Research Methodology

The study was carried out in two stages. In the preliminary tests, three trials were performed for each mixture at 100 MPa, 120 MPa, and 150 MPa pressures. After conducting the tests and analyzing the results, it was determined that satisfactory effects were obtained using the press’s maximum pressure. Therefore, the main tests were conducted at 150 MPa and a 25 mm/min feed rate. These parameters are consistent with those reported in the publication [30].

The next step was to determine the duration of the agglomeration process, the time between the end of pressing (end of pressure increase) and removal of the sample from the die, the time taken to transfer the sample to the measurement station, and the time required to set up the thermal imaging system and capture an image. This total time was 66 s for the opening die and 86 s for the closed die.

A dedicated testing station was designed to ensure proper thermographic measurements. It blocked external thermal radiation, ensured the repeatability of measurements by maintaining the same camera position, and minimized the time needed to place the test object. During the experiments, a previously designed and constructed station was used, which was also used in work [28].

The main testing procedure was carried out in the following order: weighing the proper amount of test material, placing it in the die, transferring the die to the press, setting the feed rate and pressure, starting the press, stopping it upon reaching the target pressure, and extracting the material from the die.

A FLIR T1020 thermal imaging camera (Wilsonville, OR, USA) was used to measure temperature distribution. It is equipped with a microbolometer array with a resolution of 1024 × 768 pixels and a 28° × 21° lens. Before measurement, the required parameters were set: emissivity of the tested material, ambient temperature, relative humidity, and distance to the object.

The emissivity values of the tested materials were determined experimentally using a reference tape with known emissivity. For a temperature of 49 °C (±0.1 °C), the emissivity values were as follows:

M1 (ε = 0.81), M2 (ε = 0.84), M3 (ε = 0.85), and M4 (ε = 0.69).

The thermogram analysis was performed using Thermal Studio from FLIR v. 2.0.53.

**Figure 2:**
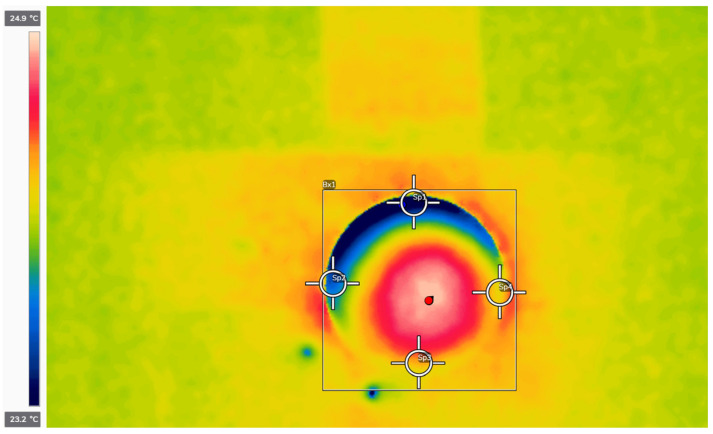
Screenshot from Thermal Studio showing a thermal image of a briquette obtained from M1 material with four measurement points.

Thermal imaging measurements were taken in various areas of the tested material. The first measurement was performed on the external surface of the briquette, recording the maximum and minimum temperatures. An example thermogram is shown in Figure 2.

During this measurement, the temperature at the center of the top surface of the briquette was also recorded. The second temperature measurement was taken at the outermost edges of the sample. The measurement points are shown in Figure 2. The final measurement was carried out inside the split briquette at various depths. On the thermal image of the split sample, five measurement points were marked: one at the top edge, one at 30% depth, one at 50% depth (midpoint), one at 70% depth, and one at the bottom edge of the tested material.

## 3. Results and Discussion

### 3.1. Measurement Results for the Opening Die

**Figure 3:**
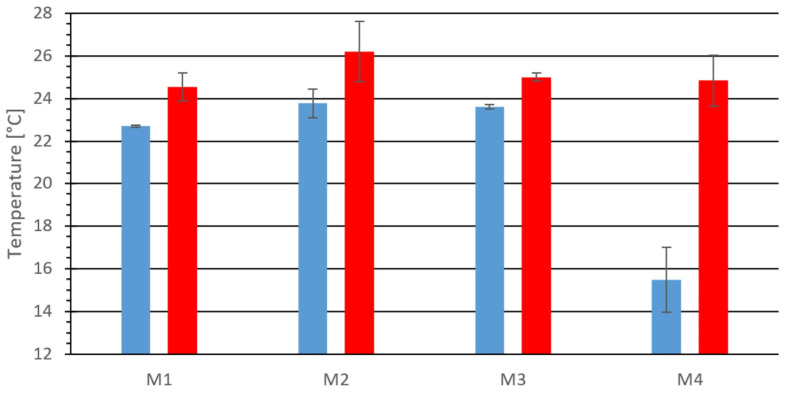
The minimum and maximum temperature on the external surface of the tested material: M1, M2, M3, and M4, blue—minimum values, red—maximum values.

Figure 3 presents the results of surface temperature measurements on the external surfaces of briquettes using the opening die.

Figure 3 compares the measured minimum and maximum temperatures for all tested mixtures. The most significant difference between the maximum and minimum temperatures was observed on the surface of material M4 (charcoal), with a difference of 9.3 °C. For the other materials, the difference ranged from 1.4 °C to 2.6 °C.

**Figure 4:**
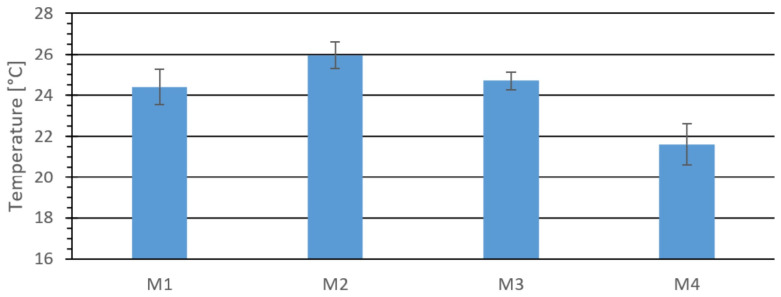
Temperature at the center of the outer upper surface of the tested material: M1, M2, M3, and M4.

Figure 4 presents the average surface temperature at the center of the top surface of the briquettes.

**Figure 5:**
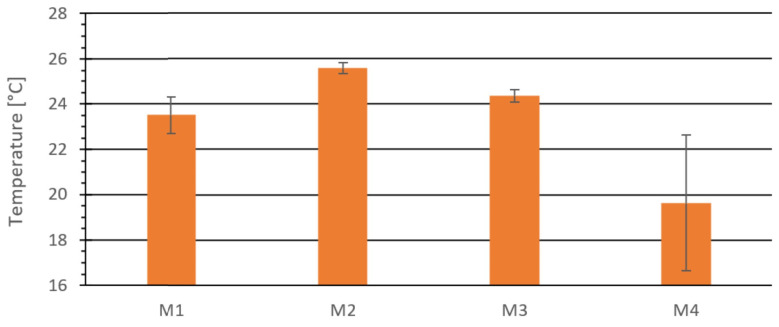
The average temperature at the extreme points of the tested material: M1, M2, M3, and M4.

Another method of visualizing the results was to show the average temperature at the outer edge surfaces of the briquettes, shown in Figure 5. The presented results are averages from three measurements.

Comparing the results in Figure 3, Figure 4 and Figure 5, it can be seen that for materials M1–M3, the highest temperature on the external surface was at the center of the top surface and the lowest at the edges. In contrast, for material M4, the highest surface temperature was not at the center. The most significant difference in surface temperature between the center and edges was 2.1 °C for material M4, and the smallest was 0.6 °C for M3. The total difference between the highest and lowest temperatures on the external surface was 9.3 °C for M4, 2.4 °C for M2, 1.9 °C for M1, and 1.4 °C for M3.

**Figure 6:**
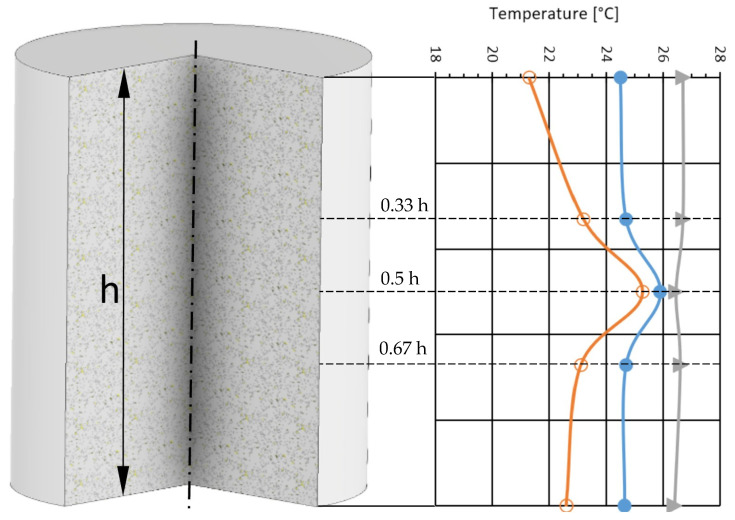
Temperature depending on the depth *g* of the measurement performed for materials M2, M3, and M4 (▲—M2, ●—M3, ○—M4).

The final step in result processing was to present the internal temperature distribution at various depths within the material. Figure 6 shows the temperature variation as a function of measurement depth for materials M2, M3, and M4.

Comparing the obtained results, it was found that for material M2, no significant difference was observed (the difference was 0.1 °C, within the measurement error). For material M3, the average internal temperature was 1.3 °C higher than the maximum surface temperature. For material M4, the difference was 2.1 °C.

From a practical point of view, for material M2, the pressure and density distribution appeared uniform across the cross-section. A similar situation was observed for material M3; the slight temperature difference suggests similar pressure values throughout the material volume. Material M4 showed the most significant temperature gradient across the cross-section, likely due to its structure and physical properties. The thermal conductivity for M2 ranges from 0.1 to 0.4 [W/(m·K)], while for M4, it ranges from 0.06 to 0.1 [W/(m·K)]. The emissivity of M4 was also the lowest among all tested materials, which may have affected the accuracy of the thermographic measurements. Materials with low emissivity tend to have larger measurement errors. This was especially visible in the wide variation in surface temperature results for M4. Material M4, consisting of 96% charcoal and 4% starch, is classified as difficult to measure using thermographic methods.

The temperature-based briquetting study aligns with the findings of other researchers. Similar research is described in publication [30], where the thermal behavior of coconut fiber was investigated for its potential use in solid biofuel (briquette) production. The briquettes were produced at 393.15 K under 15 MPa for 20 min.

### 3.2. Measurement Results for the Closed Die

**Figure 7:**
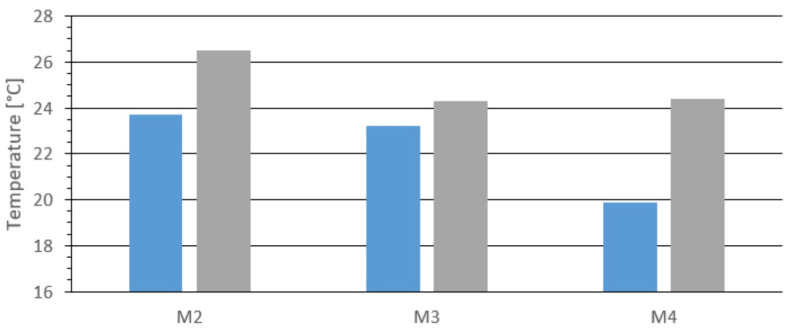
Minimum and maximum temperature on the outer surface of the briquette, blue—minimum values, grey—maximum values.

Figure 7 presents the results for the closed die, comparing the minimum and maximum temperatures measured on the surface of the tested materials. As with the opening die, the most significant temperature difference was observed for material M4 (charcoal), which was 4.5 °C. The differences between materials M2 and M3 were more minor: 2.8 °C and 1.1 °C, respectively.

**Figure 8:**
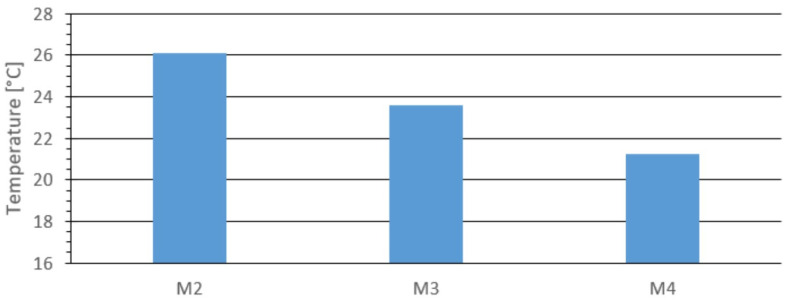
Results of the average temperature at the extreme points for the tested materials: M2, M3, and M4.

The following graph (Figure 8) shows the average temperature measured at the extreme points of the briquette surface. As in the previous analysis with the opening die, four points on the thermal image were marked and averaged. For material M4, the temperature at the briquette edges varied by approximately 1.8 °C from the average and 3.6 °C between the minimum and maximum values.

**Figure 9:**
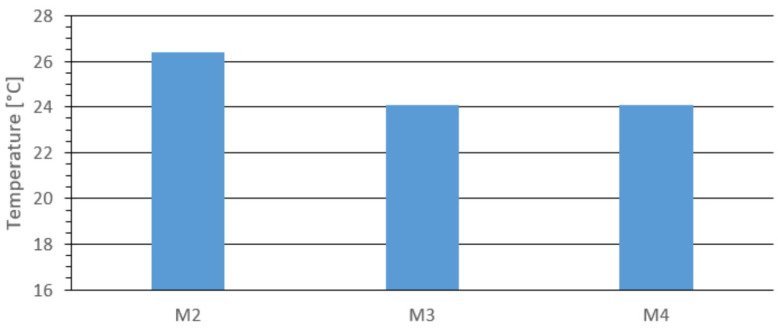
Temperature at the center of the outer upper surface for the tested materials: M2, M3, and M4.

Figure 9 shows the temperature at the center of the top surface for materials M2, M3, and M4. The highest temperature was recorded for material M2, at 26.4 °C. For the other materials, it was 24.1 °C. These results are consistent with those obtained using the opening die.

Comparing the results in Figure 7 and Figure 8, the minimum temperature for each briquette was observed at the outer edge, as with the opening die. Comparing the maximum temperature values in Figure 7 with those from the center point measurements in Figure 9, the differences were 0.1 °C for M2, 0.2 °C for M3, and 0.3 °C for M4. These minor differences fall within the expected measurement error margin.

The results suggest that the maximum temperature was located at the center of the top surface. The data are very similar, comparing results from the opening die (Figure 3, Figure 4, Figure 5, Figure 6 and Figure 7) and the closed die (Figure 7, Figure 8 and Figure 9). This supports the effectiveness of the prototype opening die. It can be concluded that the prototype ensures a proper agglomeration process for fine-grained materials. Additionally, the opening die significantly accelerates the process and allows temperature measurements to be taken immediately after agglomeration, minimizing error. The agglomeration time was 66 s for the opening die and 86 s for the closed die.

## 4. Summary

This study demonstrated that thermography can be used to analyze the briquetting processes of fine-grained materials. Each type of tested material showed a difference in surface temperature, which was either lower than or comparable to the internal temperature of the briquette. Based on the results obtained and those found in related studies, it can be concluded that stress and deformation correlate with temperature changes in briquettes (the higher the temperature in a given area, the greater the material density in that region).

An essential aspect of this work is the demonstration that thermographic testing enables continuous, non-invasive monitoring of the agglomeration process of fine-grained materials. Measuring the temperature during the agglomeration of fine-grained materials using thermography can serve as an indirect method for determining, among other things, the degree of agglomeration, the pressure generated in the forming chamber during consolidation, and the correctness of the agglomeration process.

Thermography can be used to evaluate the accuracy of the designed geometry of forming chambers, assist in selecting optimal chamber dimensions, and ensure proper execution of the agglomeration process in conditions suitable for industrial application.

The newly designed opening die has also been proven suitable for future research. The authors plan to continue the research to determine the exact relationship between the applied pressure and the local temperature of the briquette and develop a mathematical model describing the correct process of agglomerating fine-grained materials.

## Data Availability

Data are contained within the article.
